# Current Treatment of Heart Failure with Preserved Ejection Fraction

**DOI:** 10.3390/jcm14155406

**Published:** 2025-07-31

**Authors:** Mauro Riccardi, Emilia D’Elia, Carlo M. Lombardi, Gianluigi Savarese, Mauro Gori, Fabrizio Oliva, Maurizio Volterrani, Michele Senni, Marco Metra, Riccardo M. Inciardi

**Affiliations:** 1 Institute of Cardiology, ASST Spedali Civili, Department of Medical and Surgical Specialties, Radiological Sciences and Public Health, University of Brescia, 25121 Brescia, Italy; mauro94rc@hotmail.it (M.R.); lombardi.carlo@alice.it (C.M.L.); marco.metra@unibs.it (M.M.); 2 Cardiology Division, ASST Cremona, 26100 Cremona, Italy; 3 Cardiology Division, Cardiovascular Department, Papa Giovanni XXIII Hospital, 24127 Bergamo, Italy; edelia@asst-pg23.it (E.D.); mgori@asst-pg23.it (M.G.); msenni@asst-pg23.it (M.S.); 4 Division of Cardiology, Department of Clinical Science and Education, Center for Heart Failure and Arrhythmia, Karolinska University Hospital, 17100 Stockholm, Sweden; gianluigi.savarese@ki.se; 5 Cardiac Intensive Care Unit, De Gasperis Cardio Center, ASST Grande Ospedale Metropolitano Niguarda, 20162 Milan, Italy; fabrizio.oliva@ospedaleniguarda.it; 6 Department of Exercise Science and Medicine, San Raffaele Open University of Rome, 00166 Rome, Italy; maurizio.volterrani@sanraffaele.it; 7 Faculty of Medicine, University of Milano-Bicocca, ASST Papa Giovanni XXIII Hospital, 24127 Bergamo, Italy

**Keywords:** HFpEF, medical therapy, SGLT2-I, ARNI, MRA

## Abstract

Heart failure with preserved ejection fraction (HFpEF) is a heterogeneous syndrome with increasing prevalence and substantial morbidity and mortality. Recent advances in pharmacotherapy have transformed its management. This review summarizes current evidence supporting the use of sodium–glucose cotransporter 2 inhibitors, non-steroidal mineralocorticoid receptor antagonists, and glucagon-like peptide-1 receptor agonists, alongside selected use of angiotensin receptor–neprilysin inhibitors. Emphasis is placed on early initiation of disease-modifying therapies, phenotypic tailoring, and comorbidity-targeted strategies, especially in obese and diabetic patients. Together, these approaches define a new era of guideline-directed, personalized care for patients with HFpEF.

## 1. Introduction

**Figure 1:**
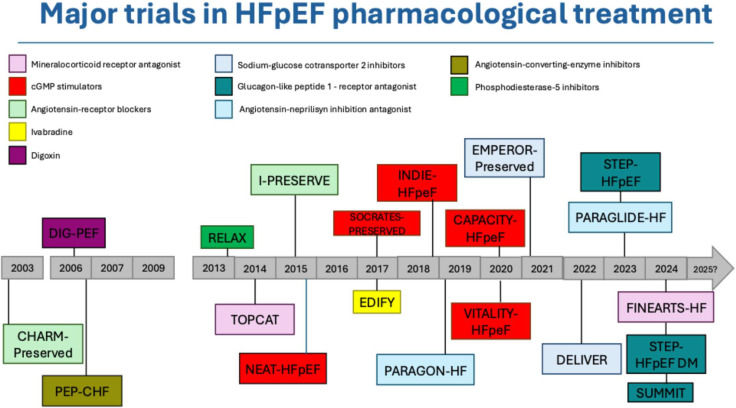
Major clinical trials in the treatment of heart failure with preserved ejection fraction.

According to universal definition, heart failure (HF) is a clinical syndrome with current or prior signs or symptoms due to structural and/or functional cardiac alterations associated with elevated natriuretic peptides or presence of pulmonary/systemic congestion [1]. Based on left ventricular (LV) ejection fraction (EF), HF with preserved EF (HFpEF) is characterized by LVEF ≥ 50%. HFpEF represents the leading cause of HF-related hospitalization worldwide [2], and its prevalence is expected to increase in parallel with the increasing age and the burden of cardiometabolic disorders [3,4]. Patients with HFpEF may be hospitalized ≥1 time per year, which contributes significantly to their high morbidity and healthcare utilization. Moreover, despite having preserved systolic function, these patients face an annual mortality of around 15%, which, while lower than in HF with reduced EF (HFrEF), is still substantial and reflects the progressive nature and high burden of disease in this population, particularly among elderly and multimorbid patients. Despite the advances in understanding the complex underlying pathophysiological mechanisms, HFpEF often remains undertreated in clinical practice. Although in the past the use of conventional medical therapy for HFrEF did not show clear benefit in HFpEF populations, treatment options have expanded in recent years [5] (Figure 1). Sodium–glucose cotransporter 2 inhibitors (SGLT2-i) are now guideline-recommended as a first-line treatment in patients with HFpEF, and sacubitril/valsartan has an indication in some countries for selected patients with HFpEF [6,7]. More recently, semaglutide, a glucagon-like peptide-1 receptor agonist (GLP1-RA), and tirzepatide, a dual GLP1 and glucose-dependent insulinotropic polypeptide (GIP) receptor agonist, have been shown to improve health status and body weight in patients with HFpEF and obesity [8,9]. Lastly, the non-steroidal mineralocorticoid receptor antagonist finerenone recently showed to improve mortality/morbidity in a wide category of patients which also included those with HFpEF.

The recent progress in HFpEF pharmacological therapy, coupled with the substantial residual risk in this population, underscores the need for an accelerated optimization of foundational medical therapy. This review will summarize the main findings about this topic. We will exclude treatment of specific causes of HFpEF, such as cardiac amyloidosis or hypertrophic cardiomyopathy as these conditions deserve specific therapies and their consideration goes beyond the aims of the present article.

## 2. Sodium–Glucose Cotransporter-2 Inhibitors as First Pillar of HFpEF Treatment

First-line therapy for HFpEF includes SGLT2-i empagliflozin and dapagliflozin, which have shown consistent cardiovascular (CV) benefit, health status improvement, and safety (Table 1 and Table 2). Although sacubitril/valsartan was evaluated earlier in HFpEF trials, we refer to SGLT2-i as the first pillar of HFpEF therapy because they are currently the only pharmacological class with a class I recommendation in the 2023 European HF Guidelines for patients with HFpEF [10]. In the “Empagliflozin Outcome Trial in Patients with Chronic Heart Failure with Preserved Ejection Fraction (EMPEROR-Preserved)” [11] and in “Dapagliflozin Evaluation to Improve the LIVEs of Patients With PReserved Ejection Fraction Heart Failure (DELIVER)” [12], both empagliflozin and dapagliflozin reduced the combined risk of CV death or hospitalization for HF of 21% and 18%, respectively, regardless of the presence of type 2 diabetes mellitus (T2DM), among HF patients with LVEF > 40%. In both trials, this effect was mainly related to a lower risk of hospitalization for HF (−27% and −21%) while no statistically significant reduction in CV mortality was observed in either trial. Also including HF with mildly reduced ejection fraction (HFmrEF) patients, subgroup analyses helped to clarify the benefit specifically in HFpEF patients. In the DELIVER trial, dapagliflozin reduced the composite outcome both in patients with LVEF between 50 and 59% (HR 0.79, 95% CI 0.65–0.97) and in those with LVEF ≥ 60% (HR 0.78, 95% CI 0.62–0.98). Similarly, in the EMPEROR-Preserved trial, empagliflozin significantly reduced the composite outcome in patients with LVEF between 50 and 59% (HR 0.80, 95% CI 0.64–0.99), while the effect was attenuated in those with LVEF ≥ 60% (HR 0.87, 95% CI 0.69–1.10). These results suggest that the clinical benefit of SGLT2-i is largely consistent in patients across the higher end of the LVEF spectrum, including those meeting criteria for HFpEF. It is noteworthy that in the DELIVER trial, the CV benefit was consistent also among patients with a history of improved LVEF.

In a pooled meta-analysis including 12,251 participants from DELIVER and EMPEROR-Preserved [13], SGLT2-i led to a 20% reduction in CV death or first hospitalization with consistent reductions in individual components (12% risk reduction in CV death and 26% risk reduction in first hospitalization for HF).

There was also a modest significant improvement in quality-of-life scores with both agents, with an increase in the Kansas City Cardiomyopathy Questionnaire (KCCQ) of 1.32 points with empagliflozin and 1.11 points with dapagliflozin.

Prespecified analysis from DELIVER and EMPEROR-Preserved showed a statistically significant reductions in the primary end point observed within 20 days of randomization [14,15].

SGLT2-i also showed consistent benefit regardless of the timing of the most recent HF hospitalization supporting its early use in patients hospitalized for HF. In the “Empagliflozin in Patients Hospitalized With Acute Heart Failure Who Have Been Stabilized (EMPULSE)” trial, treatment with empagliflozin was well-tolerated and led to more rapid and effective decongestion in the acute HF setting and was also associated with significant improvement of a composite endpoint including clinical outcome (death, HF events) and health status in the 22% of patients with HFpEF [16,17,18]. Similarly, in the “Efficacy and Safety of Dapagliflozin in Acute Heart Failure (DICTATE-AHF)”, dapagliflozin increased diuretic efficiency and cumulative diuresis, also allowing an earlier discontinuation of diuretics during hospitalization [19]. The effect of dapagliflozin on the primary outcome was generally consistent across prespecified subgroups, including the 43% of patients with HFpEF. In the “Effect of Sotagliflozin on Cardiovascular Events in Patients with Type 2 Diabetes Post Worsening Heart Failure trial (SOLOIST-WHF)” study, sotagliflozin initiation among patients with T2DM and recent worsening HF resulted in a significantly lower total number of CV death and hospitalizations and urgent visits for HF than placebo regardless of baseline LVEF below or above 50% [19].

The consistent safety and tolerability of SGLT2-i have been confirmed in the meta-analysis from DELIVER and EMPEROR-Preserved, where any serious adverse event occurred numerically less frequently in the SGLT2-i groups compared with the placebo groups in both trials [13] (Table 2). Similarly, the rates of most adverse events were infrequent and similar, with the possible exception of genito-urinary tract infections, between SGLT2i and placebo groups. Taken together, the available evidence on SGLT2 inhibitors in HFpEF support the early adoption of this class of drug as a first-line treatment, highlighting key opportunities for the prompt management of this patient population without delay [20].

## 3. Angiotensin Receptor–Neprilysin Inhibitors as an Integrated Therapy in Specific Settings

In the “Angiotensin-Neprilysin Inhibition in Heart Failure with Preserved Ejection Fraction (PARAGON-HF)” [21], the primary composite endpoint of total hospitalizations for HF and CV death was only numerically lower with sacubitril/valsartan (12.8 events per 100 patient-years vs. 14.6 events per 100 patient-years; HR 0.87, 95% CI 0.75–1.01) among HFpEF patients (LVEF ≥ 45%).

Prespecified analysis showed a significant risk reduction in women compared to men (*p* for interaction = 0.017) with a benefit mostly attributable to HF hospitalization reduction [22]. However, the PARAGON-HF trial was not specifically powered to detect sex-based differences in treatment effect. Moreover, potential benefit was also observed in the lower rate of preserved ejection fraction (LVEF ≤ 57%) (HR 0.78, 95% CI 0.64–0.95). Additionally, a subgroup analysis revealed a significant reduction in HF hospitalizations in patients with an eGFR < 60 mL/min/1.73 m^2^ (HR 0.81, 95% CI 0.68–0.96; *p* = 0.027), underscoring the potential benefit of sacubitril/valsartan in individuals with renal dysfunction.

The efficacy and safety of sacubitril/valsartan has also been recently explored among HFpEF patients recently hospitalized or in a worsening HF setting. The results of the “Prospective comparison of ARNI with ARB Given following stabiLization In DEcompensated HFpEF (PARAGLIDE-HF)” study [23] showed a trend favoring sacubitril/valsartan vs. valsartan to reduce a hierarchical clinical endpoint of CV death, HF hospitalizations, urgent HF visits, and change in NT-proBNP among HFpEF patients (LVEF > 40%) with recent worsening HF. Benefits were more evident in patients with LVEF ≤ 60% (HR 0.78, 95% CI 0.65–0.93) compared to patients with LVEF > 60% (HR 1.17, 95% CI 0.86–1.59, *p* for interaction = 0.033).

The pooled analysis of PARAGON-HF and PARAGLIDE-HF showed that sacubitril/valsartan significantly reduced the primary composite endpoint of worsening HF events (including first and recurrent HF hospitalization) and CV death compared with valsartan (RR 0.86; 95% CI, 0.75–0.98) (Table 1). The analysis confirmed previous observations of treatment heterogeneity by LVEF as treatment benefits were larger in those with LVEF ≤ 60% compared with those with LVEF > 60% (*p* for interaction = 0.021) [24].

Based on the results of PARAGON-HF, sacubitril/valsartan received indications for use in selected patients with HFpEF and LVEF in the lower range of preserved spectrum in the USA (class IIb recommendation) and other countries while European guidelines do not assign a specific class of recommendation for this population, reflecting ongoing uncertainties.

## 4. Glucagon-Like Peptide-1 Receptor Agonist as a Tailored Therapy for Obese HFpEF

Over the past years, increasing evidence has highlighted the therapeutic potential of GLP1-RAs in managing obesity and reducing CV risk. In large randomized clinical trials, GLP1-RAs were associated with a 12% lower risk of HF hospitalization among individuals with T2DM [25]. More recently, their role has expanded following the publication of positive trials evaluating semaglutide in both diabetic and non-diabetic obese patients (body mass index ≥ 30 kg/m^2^) with HFpEF (LVEF ≥ 45%) (Table 1).

In the “Semaglutide in Patients with Heart Failure with Preserved Ejection Fraction and Obesity (STEP-HFpEF)” study, semaglutide led to larger reductions in health-related quality of life (estimated KCCQ-CCS difference 7.8 points), greater improvements in exercise function (estimated six-minute walking distance difference 20.3 m), greater weight loss (estimated difference −10.7% points), and, importantly, greater reduction in NT-proBNP levels than placebo [26]. Similarly, in the “Semaglutide in Patients with Obesity-Related Heart Failure and Type 2 Diabetes (STEP-HFpEF DM)” study [27], semaglutide showed a larger reduction in health-related quality of life (estimated KCCQ-CCS difference 7.3 points), greater improvements in exercise function (estimated six-minute walk distance change 14.3 m), greater weight loss (estimated difference −6.4% points), and greater reduction in NT-proBNP levels than placebo. In the pooled analysis [8], semaglutide led to a significant improvement in HF–related symptoms (7.5 points estimated treatment difference) across the entire LVEF spectrum (*p* for interaction = 0.55) and was associated with a body weight reduction of ~8%. Although the trials were not specifically powered to assess clinical events, there were fewer HF hospitalizations or urgent visits for HF among the semaglutide-treated patients compared with placebo (HR 0.27, 95% CI 0.12–0.56). The safety profile was favorable, with fewer serious adverse events, cardiac complications, and infections reported in the semaglutide group. Gastrointestinal (GI) side effects that led to discontinuation were more frequently noted with semaglutide; however, the incidence of serious GI events, including pancreatitis, was comparable between treatment arms (Table 2).

It has been recently questioned to what extent semaglutide can improve HF pathophysiology beyond body weight reduction. A recent analysis from the STEP-HFpEF program showed that semaglutide compared with placebo reduced NT-proBNP at 52 weeks (estimated treatment ratio: 0.82; 95% CI: 0.74–0.91; *p* = 0.0002) and participants with higher baseline NT-proBNP had a similar degree of weight loss but experienced larger reductions in HF-related symptoms and physical limitations with semaglutide than those with lower NT-proBNP [28]. These benefits were more pronounced among patients with atrial fibrillation at baseline [29] and receiving loop diuretics [30]. Moreover, semaglutide also led to a reduction in loop diuretic use and dose between baseline and 52 weeks [30]. Lastly, in a pooled analysis of the SELECT, FLOW, STEP-HFpEF, and STEP-HFpEF DM randomized trials, among patients with HFpEF (n = 3743), semaglutide reduced the risk of the combined endpoint of CV death or worsening HF events, and worsening HF events alone, whereas its effect on CV death alone was less pronounced [31]. Semaglutide has also been associated with favorable effects on cardiac structure and function, including reductions in left atrial volume, improvements in LV diastolic function, and decreased right ventricular dimensions [28]. These findings collectively suggest that the observed HF benefits of GLP1-RA are unlikely to be simply related to weight loss but underlie specific disease-modifying effects, weight loss-independent. Indeed, semaglutide has been shown to reduce the degree of inflammation, a key feature of patients with HFpEF and obesity [32]. It is acknowledged, however, that maintaining full blinding in these trials may be challenging due to the marked weight loss observed in a significant proportion of patients. Further evidence on the beneficial role of this class of drug derive from the dual agonist of the GIP and GLP1-RA tirzepatide. The combination of GIP and GLP-1 receptor activation appears to have additive and synergistic effects on glycemic control, weight reduction, and potential cardiovascular benefits compared to GLP-1 receptor agonism alone. The “Study of Tirzepatide in Participants With Heart Failure With Preserved Ejection Fraction and Obesity (SUMMIT)” trial demonstrated a 38% risk reduction in HF (HF urgent visit or hospitalization, oral diuretic intensification or CV death) and a 15.7% body weight reduction with improvement in health status among patients with obesity and HFpEF [9,33]. A reduction in the degree of inflammation has also been found, as already reported for semaglutide [34]. From a pathophysiological perspective, a CMR sub-study of the SUMMIT trial showed that tirzepatide reduced LV mass and paracardiac adipose tissue as compared with placebo, potentially explaining the reduction in heart failure events observed in the trial [35]. Overall, these findings suggest an early use of GLP1-RA among obese HFpEF patients. Although more data on hard clinical endpoints with larger population are needed, this treatment regimen should represent the standard of care for obese patients with evidence of HF to improve health status and body weight.

## 5. Non-Steroidal Mineralocorticoid Receptor Antagonists as a New Frontier in HFpEF Treatment

Mineralocorticoid receptor antagonists (MRA) represent a foundational therapy for HFrEF. However, the benefit of MRAs is less well established in patients with HF at higher LVEF. In the “Treatment of Preserved Cardiac Function Heart Failure with an Aldosterone Antagonist TOPCAT)” [36], spironolactone failed to show benefit with regard to CV death or HF hospitalization, although post hoc analyses suggested potential benefit among patients enrolled in the Americas and misconduct in Georgia/Russia which might contribute to explain the results.

While US guidelines provide weak recommendation for its use in HFpEF, European guidelines do not assign a specific class of recommendation. In addition, the broad use of steroidal MRA has been limited overall, even in HFrEF, due to the risk of renal dysfunction and hyperkalaemia.

Recently, the non-steroidal MRA finerenone has been developed to have higher selectivity for the mineralocorticoid receptor than steroidal MRAs and a balanced tissue distribution in the heart and kidneys, thus limiting risks of electrolyte disturbances.

Two large-scale phase 3 clinical trials showed that finerenone is safe and effective in slowing kidney disease progression and preventing CV events in over 13,000 patients with chronic kidney disease and T2DM [37]. Although HFrEF patients were excluded, finerenone significantly reduced risks of hospitalization for HF by 22%. The “FINerenone trial to investigate Efficacy and sAfety superioR to placebo in paTientS with Heart Failure (FINEARTS-HF)” trial has been recently designed to evaluate the long-term efficacy and safety of finerenone among patients with HF with LVEF ≥ 40% [38]. The study showed a reduction in a composite of total worsening HF events and death from CV causes in patients treated with finerenone compared to placebo (risk ratio 0.84; 95% confidence interval, 0.74–0.95; *p* = 0.007), driven by a significant reduction in HF events [38,39]. Long-term treatment was estimated to extend event-free survival by up to 3 years [40]. Results were consistent regardless of baseline LVEF (LVEF < 50% RR 0.84, 95% CI 0.68–1.03; LVEF ≥ 50% to <60% RR 0.80, 95% CI 0.66–0.97; and LVEF ≥ 60% RR 0.94, 95% CI 0.70–1.25; *p* for interaction = 0.70) [41], background use of SGLT2-I, and sex [42]. Finerenone also resulted in a moderate benefit with respect to improvement in health-related quality of life (estimate KCCQ-CCS difference 1.6 points). With respect to safety and tolerability, the incidence of serious adverse events was overall similar in the two groups, although elevated creatinine levels and hyperkalaemia occurred more frequently in the finerenone group than in the placebo group (Table 2). However, rate of hyperkalemia leading to hospitalization were similar in the two groups, and no patients died for hyperkalemia in the trial. Furthermore, although finerenone led to a higher initial estimated glomerular filtration rate (eGFR) reduction, this did not translate into a significant difference in chronic eGFR slope, vs. placebo. Early and sustained reductions in albuminuria were also observed [43,44].

A prespecified analysis of the FINEARTS-HF trial demonstrated that finerenone reduced the primary outcome similarly regardless of baseline use of SGLT2 inhibitors (*p* for interaction = 0.76) and that the introduction of SGLT2 inhibitors during follow-up did not significantly modify the treatment effect in a “time-updated” analysis. The authors therefore suggested that the combined use of SGLT2 inhibitors and a non-steroidal MRA could provide additive and synergistic protection in terms of outcomes [45]. Recent data also showed that the benefit of finerenone appeared to decrease after short-term drug withdrawal. In addition, the rate of CV serious adverse events increased after discontinuation of finerenone but not placebo.

Taken together, the results of FINEARTS-HF provide new evidence for implementation of medical therapy in HFpEF patients, and non-steroidal MRA finerenone should be considered as a foundational therapy in this population.

## 6. Upfront Treatment Approach for HFpEF Patients

**Figure 2:**
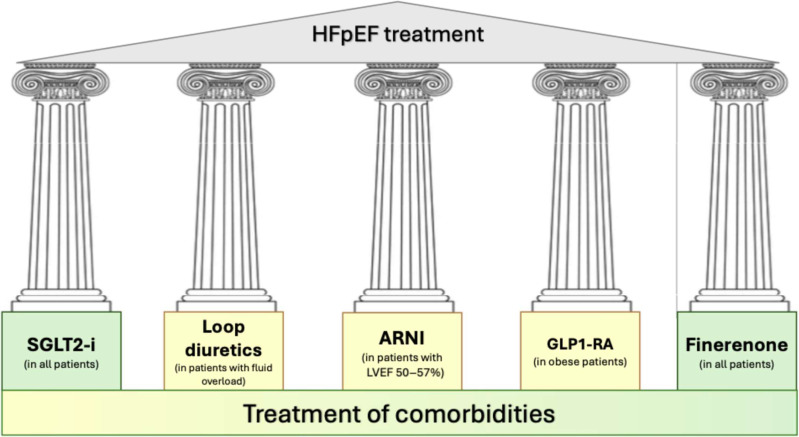
HFpEF treatment approach. Patients with HFpEF have a significant burden of cardiac- and non- cardiac comorbidities. The identification and treatment of underlying comorbidities is of critical importance. SGLT2-i and non-steroidal MRA finerenone should be considered as first-line therapy, regardless of the phenotype and the clinical settings, for all HFpEF patients lacking contraindications. ARNI therapy should be considered in all patients with HFpEF and an LVEF between 50% and 57%, as well as in those with a recent episode of worsening HF. In individuals with HFpEF and obesity, irrespective of diabetic status, treatment with a GLP1-RA should be initiated to promote weight reduction and improve overall health status. Diuretics remain essential for symptom relief in patients with clinical congestion but should be administered with careful attention to volume status and renal function.

Clinical management of HFpEF has become increasingly nuanced in the context of expanding therapeutic options. In routine practice, a holistic strategy that integrates all evidence-based, life-prolonging interventions should serve as the foundation of HFpEF care (Figure 2). Concurrently, these patients often present with a high prevalence of both CV and extracardiac comorbidities, including obesity, hypertension, T2DM, coronary artery disease, and atrial fibrillation, that further complicate treatment planning. Optimal control of blood pressure and glycemia, rhythm or rate management in atrial fibrillation, and ischemia-directed therapies are essential to improve outcomes. As such, the identification and treatment of underlying comorbidities is of critical importance for a comprehensive management of HFpEF. Tailoring pharmacologic therapy based on the underlying HFpEF phenotype may enable a more individualized and effective treatment strategy.

SGLT2-i dapagliflozin and empagliflozin should be considered as first-line therapy regardless of the phenotype and the clinical settings for all HFpEF patients lacking contraindications. The non-steroidal MRA finerenone may serve as a second cornerstone therapy for HFpEF. Based on findings from the FINEARTS-HF trial, early initiation of finerenone, potentially in close sequence with an SGLT2-I, could be considered. ARNI therapy should be reserved for patients with HFpEF and an LVEF between 50% and 57%, or for those who have recently experienced a worsening HF episode. More broadly, both ARNI and MRA therapies should be maintained in patients with a history of HF and recovered or improved LVEF.

Among obese HFpEF, regardless of the diabetic status, GLP1-RA should be started to improve health status and manage body weight. Diuretic therapy should be used judiciously for patients with overt congestion and includes loop diuretics, which reduces cardiac filling pressures and favors decongestion. Finally, beta-blockers may be used in individuals with HFpEF who have specific indications, such as atrial fibrillation, but exercise tolerance should be monitored due to the potential for chronotropic incompetence [46].

However, while emerging therapies have opened new horizons for HFpEF treatment, it is important to acknowledge that, unlike HFrEF, the absolute benefits in HFpEF are less pronounced for hard outcomes. Most large trials did not demonstrate significant mortality reductions, while providing efficacy in HF hospitalizations. Other trials, such as those with semaglutide, were not designed to test hard outcomes. Improvements in quality-of-life scores, although statistically significant, often fall below established thresholds of clinical meaningfulness. Nonetheless, in a predominantly elderly, multimorbid population, even small gains in symptom burden and reduced HF events may translate into real-world benefit, particularly when treatment is safe and well-tolerated. As recently discussed by Kondo et al., the absence of demonstrated mortality benefit in HFpEF likely reflects a combination of biological heterogeneity, competing non-CV risks, and limitations in trial design and patient selection [47].

Future studies with better phenotypic matching may enhance treatment efficacy, but current data support a cautious but optimistic move toward a more structured, multi-therapy approach.

## 7. Future Perspectives

Despite the recent treatment success, patients with HFpEF experience a significant residual risk of hospitalization and mortality [3]. For this reason, it is critical to develop new therapeutic strategies to improve the care of this population. As shown in Table 3, ongoing clinical trials are testing new treatment regimens. The ultimate goal is to develop therapeutic options both acting for to all HF patients and targeted on HFpEF phenotypes. Recent evidence suggests a potential beneficial effect of ferric carboxymaltose in patients with HFpEF and evidence of iron deficiency in terms of improvement in a 6 min walking test distance. However, larger trials are needed to establish whether intravenous iron can improve quality of life and eventually CV outcomes in patients with HFpEF [48].

Given the burden of inflammation characterizing HFpEF patients, the “Effects of Ziltivekimab Versus Placebo on Morbidity and Mortality in Patients With Heart Failure With Mildly Reduced or Preserved Ejection Fraction and Systemic Inflammation (HERMES)” trial (NCT05636176), an ongoing multicenter, randomized, double-blind study, is evaluating the effect of ziltivekimab, a monoclonal antibody targeting the IL-6 ligand, compared with placebo on the primary composite outcome of time to first occurrence of CV death, HF hospitalization or urgent HF visit in patients with HFpEF and HFmrEF. In addition, with the aim of reducing inflammation, other trials are evaluating the role of low-dose colchicine, already used in acute and chronic coronary syndromes [49,50], in patients with HFpEF (NCT06130059 and NCT05637398).

Coronary microvascular endothelial inflammation and myocardial fibrosis are two other mechanisms involved in the pathophysiology of HFpEF [3]. Pirfenidone is a drug with antifibrotic and anti-inflammatory properties that has been shown to have cardioprotective effects. The “Pirfenidone in Patients with Heart Failure and Preserved Left Ventricular Ejection Fraction (PIROUETTE)” study assessed the efficacy and safety of pirfenidone in 94 HFpEF (LVEF > 45%) patients with myocardial fibrosis at cardiac magnetic resonance examination. Results showed that pirfenidone treatment reduced myocardial extracellular volume and NT-proBNP values. The most common adverse events of pirfenidone included nausea, insomnia, and rash. Further trials are necessary to determine the clinical effectiveness and safety of pirfenidone in HFpEF.

Ongoing upcoming trials on steroidal MRA (NCT02901184, NCT04727073) will further clarify the role of MRA on HFpEF treatment and might help form an understanding of whether the CV benefit relies upon a class effect or not. The clinical trial EASi-HF (NCT05686970) is a phase III, randomized, double-blind, placebo-controlled study designed to evaluate the efficacy and safety of the combination of vicadrostat (previously known as BI 690517) and empagliflozin in patients with symptomatic HF and an LVEF ≥ 40%.

In an open-label trial in patients with HFpEF with an LVEF greater than 60%, mavacamten was associated with improvements in NT-proBNP and cardiac troponins with no sustained LVEF reductions [51]. A phase 2 randomized clinical trial of a next-generation cardiac myosin inhibitor is currently underway (NCT06122779) to further validate and expand upon these findings.

Lastly, two ongoing trials (NCT04945707 and NCT05991128) will evaluate the impact of intravenous ferric derisomaltose, previously tested in patients with an LVEF < 45% [52], on exercise capacity in HFpEF patients.

## 8. Conclusions

The therapeutic landscape for HFpEF is currently undergoing a transformational change. Novel therapeutic options including SGLT2-i, non-steroidal MRA, and GLP1-RA advocate a paradigm shift in the care of HFpEF patients. An upfront combination of foundational therapies should represent the gold-standard of HFpEF treatment by targeting multiple pathophysiological drivers. A simultaneous or early sequential initiation of SGLT2-i and finerenone should be advocated, complemented by phenotype-guided therapies such as GLP1-RA and ARNI, depending on the clinical context. This integrated, patient-centered strategy is essential to improve quality of life and clinical outcomes in individuals with HFpEF, ultimately supporting clinicians in delivering more effective and personalized care.

## Figures and Tables

**Table 1:** Major positive randomized controlled trial in HFpEF.

	DELIVER	EMPEROR-Preserved	Pooled PARAGON-HF/PARAGLIDE-HF	STEP-HFpEF	STEP-HFpEF DM	FINEARTS-HF	SUMMIT
Size	N = 6263	N = 5988	N = 5262	N = 529	N = 616	N = 6001	N = 731
Agent	Dapagliflozin	Empagliflozin	Sacubitril/valsartan	Semaglutide	Semaglutide	Finerenone	Tirzepatide
Age, year	72	72	72	69	69	72	65
Baseline EF (%)	54	54	57	57	56	53	61
Primary outcome	Worsening HF and CV death: HR 0.82 (95% CI 0.73–0.92)	Hospitalization for HF and CV death: HR: 0.79 (95% CI 0.69–0.90)	Total hospitalizations for HF and CV death: RR 0.86 (95% CI 0.75–0.98)	Change in KCCQ-CSS (difference 7.8 points; 95% CI 4.8–10.9) and change in body weight (difference −10.7%, 95% CI −11.9–−9.4)	Change in KCCQ-CSS (difference 7.3 points; 95% CI 4.1–10.4) and change in body weight (difference −6.4%, 95% CI −7.6–−5.2)	Total worsening HF events and CV death: RR 0.84 (95% CI 0.74–0.95)	A composite of death from cardiovascular causes or a worsening heart-failure event, and the change at 52 weeks in the KCCQ-CSS
Hospitalization for HF	HR 0.77 (95% CI 0.67–0.89)	HR 0.71 (95% CI 0.60–0.83)	-	HR 0.08 (95% CI 0.00–0.42)	HR 0.40 (95% CI 0.15–0.92	RR 0.82 (95% CI 0.71–0.94)	HR 0.44 (95% CI 0.22–0.87)
CV death	HR 0.88 (95% CI 0.74–1.05)	HR 0.91 (95% CI 0.76–1.09)	HR 0.93 (95% CI 0.77–1.12)	-	-	HR 0.93 (95% CI 0.78–1.11)	HR 1.58 (95% CI 0.52–4.83)

**Table 2:** Adverse events in major HFpEF clinical trials.

Abbreviations: CI, Confidence Interval; CV, Cardiovascular; EF, Ejection Fraction; HF, Heart Failure; HR, Hazard ratio; KCCQ-CSS, Kansan City Cardiomyopathy Questionnaire—Clinical Summary Score; NR, Not Reported; RR, Rate Ratio.	DELIVER (Dapagliflozin vs. Placebo)	EMPEROR-Preserved (Empagliflozin vs. Placebo)	Pooled PARAGON-HF/ PARAGLIDE-HF (Sacubitril/Valsartan vs. Valsartan)	STEP-HFpEF (Semaglutide vs. Placebo)	STEP-HFpEF DM (Semaglutide vs. Placebo)	FINEARTS-HF (Finerenone vs. Placebo)	SUMMIT (Tirzepatide vs. Placebo)
Any Serious AEs (%)	43.5 vs. 45.5	47.9 vs. 51.6	-	13.3 vs. 26.7	17.7 vs. 28.8	38.7 vs. 40.5	26.4 vs. 25.6
Hypotension	-	6.6 vs. 5.2	23.4 vs. 16.9	-	-	18.5 vs. 12.4	6.0 vs. 3.0
Renal adverse events	2.3 vs. 2.5	12.1 vs. 12.8	2.2 vs. 3.7	2.3 vs. 1.5	0.6 vs. 2.6	2.0 vs. 1.2	1.4 vs. 0.8
Hypoglycemic events	-	2.4 vs. 2.6	-	-	-	-	
Hyperkaliemia (≥5.5 mEq/L)	-	-	18.5 vs. 18.1	-	-	14.3 vs. 6.9	
Lower Limb Amputation	-	0.5 vs. 0.8	-	-	-	-	
Gastrointestinal disorder	-	-	-	2.7 vs. 2.6	1.6 vs. 1.6	-	Diarrhea 18.6 vs. 6.3 Vomiting 10.4 vs. 2.2
Urinary tract infections	1 vs. 1	9.9 vs. 8.1	-	-	-	-	1.4 vs. 0.3
Genital infections	-	2.2 vs. 0.7	-	-	-	-	-
AEs leading to treatment discontinuation (%)	5.8 vs. 5.8	19.1 vs. 18.4	-	2.3 vs. 2.3	1.9 vs. 3.6	-	6.3 vs. 1.4

**Table 3:** Ongoing clinical trials with medical therapy in individuals with HFpEF.

Trial	Registration Number	Trial Design	Arms	Estimate Enrolment	Primary Endpoint
SPIRIT-HF	NCT04727073	Randomized	Spironolactone vs. Placebo	N = 1300	Cumulative number of primary composite events of CV death and total HFH
SPIRRIT-HFpEF	NCT02901184	Randomized	Spironolactone vs. standard care	N = 2000	Incidence rate for total HFH or CV death
HERMES	NCT06200207	Randomized	Ziltivekimab vs. Placebo	N = 680	A composite of CV death, HFH or urgent HF visit
RENEU-HF	NCT06369298	Randomized	JK07 vs. Placebo	N = 282	Safety and efficacy
CARE-Preserved HF	NCT05553314	Randomized	Carvedilol vs. Placebo	N = 100	Change in NT-proBNP and GLS
CADENCE	NCT04945460	Randomized	Sotatercept vs. Placebo	N = 150	Change in PVR
IMPROVE-DiCE	NCT04826159	Single-group	IMB-1018972	N = 50	Cardiac energetic reserve at rest and during stress
AURORA-HFpEF	NCT06122779	Randomized	BMS-986435 vs. Placebo	N = 48	Safety and tolerability
SOTA-P-CARDIA	NCT05562063	Randomized	Sotagliflozin vs. Placebo	N = 100	Change in LV mass in cardiac magnetic resonance
Low Dose Colchicine among Patients With Chronic Stable HFpEF and Systemic Inflammation	NCT06130059	Randomized	Low-dose colchicine vs. Placebo	N = 60	Change in VO_2_ peak indexed to body weight
Colchicine in Patients With Heart Failure and Preserved Left Ventricular Ejection Fraction	NCT05637398	Randomized	Colchicine vs. Placebo	N = 40	Change in sST2
Efficacy and Safety of LY3540378 in Adults With Worsening Chronic Heart Failure With Preserved Ejection Fraction	NCT05592275	Randomized	LY3540378 vs. Placebo	N = 432	Change in LA Reservoir Strain
IRONMET HFpEF	NCT04945707	Randomized	Ferric Derisomaltose vs. Placebo	N = 66	Change in VO_2_ peak
COREVIVE-HFpEF	NCT05991128	Randomized	Ferric Derisomaltose vs. Placebo	N = 170	Change in 6MWD
Cycle-2-PEF	NCT06215586	Randomized	Tovinontrine vs. Placebo	N = 240	Change in NT-proBNP
INABLE-Training	NCT02713126	Randomized	Sodium nitrite vs. placebo	N = 92	Change in VO_2_ peak
KNO3CK OUT HFpEF	NCT02840799	Randomized	Potassium nitrate vs. potassium chloride	N = 84	Change in VO_2_ peak
EASi-HF	NCT06424288	Randomized	Vicadrostat + empagliflozin vs. placebo	N = 6000	Time to first event of CV death or HFH

Abbreviations: CV—cardiovascular; GLS—global longitudinal strain; HF—heart failure; HFH—heart failure hospitalizations; HFpEF—heart failure with preserved ejection fraction; KCCQ-CSS—Kansan City Cardiomyopathy Questionnaire—clinical summary score; LA—left atrial; LV—left ventricular; PVR—pulmonary vascular resistance; 6MWD—six-minute walking distance.

## Data Availability

No new data were created or analyzed in this study.

## Abbreviations

AEs—adverse events.
